# Polymorphism of *CONNEXIN37* gene is a risk factor for ischemic stroke in Han Chinese population

**DOI:** 10.1186/s12944-018-0727-3

**Published:** 2018-04-10

**Authors:** Hong Li, Shasha Yu, Rui Wang, Zhaoqing Sun, Xinghu Zhou, Liqiang Zheng, Zhihua Yin, Yingxian Sun

**Affiliations:** 1 Department of Cardiology, Shengjing Hospital of China Medical University, Shenyang, 110004 China; 2 Department of Cardiology, the First Hospital of China Medical University, 155 North Nanjing Street, Shenyang, 110001 China; 3 Department of Clinical Epidemiology, Library, Shengjing Hospital of China Medical University, Shenyang, 110004 China; 4 Epidemiology Department of China Medical University, Shenyang, 110122 China

**Keywords:** Stroke, *connexin37*, SNPs, Han Chinese, Gene polymorphism

## Abstract

**Background:**

Stroke has a high fatality and disability rate, and is one of the main burdens to human health. It is thus very important to identify biomarkers for the development of effective approaches for the prevention and treatment of stroke. Connexin37 is an anti-inflammatory cytokine and is involved in chronic inflammation and atherosclerosis. Recent studies have found that *CONNEXIN37* gene variations are associated with atherosclerosis diseases, such as coronary heart disease and stroke, but its association with stroke in distinct human populations remains to be determined. We report here the analysis of the association of the single nucleotide polymorphisms (SNPs) of *CONNEXIN37* with ischemic stroke in Han Chinese population.

**Methods:**

Two SNPs of *CONNEXIN37* gene were analyzed in 385 ischemic stroke patients and 362 hypertension control patients using ligase detection reaction (LDR) method.

**Results:**

Logistic regression analysis demonstrated that, AG and GG genotypes of SNP rs1764390 and CC genotype of rs1764391 of *CONNEXIN37* were associated with an increased risk of ischemic stroke, and that G allele of rs1764390 is a risk factor for ischemic stroke. Further, we found that SNP rs1764390 and SNP rs1764391 in *CONNEXIN37* were associated with ischemic stroke under additive/dominant model, and recessive/dominant model, respectively.

**Conclusion:**

Our results indicate that *CONNEXIN37* gene polymorphism is an ischemic stroke risk factor in Northern Han Chinese.

## Background

Stroke is a complex disease with very high mortality and morbidity [1]. Age, gender, ethnicity, hypertension, diabetes, hyperlipidemia, obesity, smoking, poor eating habits and others have been shown to be correlated with stroke [1, 2]. Recent evidences have suggested that the genetic background of an individual contributes to stroke incidence and prognosis [2–6]. Moreover, genetic factors may affect stroke via distinct mechanisms [2, 7], and an individual with a certain gene polymorphism may be more susceptible to stroke and resistant to drug intervention [8–10]. Therefore, gene polymorphism might be an independent risk factor for stroke, suggesting the importance of the identification and validation of biomarkers for the diagnosis, treatment and prognosis of stroke in the era of precision medicine.

Coagulation/fibrinolytic system-related genes, lipoprotein gene, renin-angiotensin system (*ACE*) related gene, endothelial nitric oxide synthase (*NO*), methylenetetrahydrofolate reductase (*MTHFR*), brain natriuretic peptide (*BNP*), protein kinase C (*PKC*), *PDE4D*, and *ALOX5AP* have been found to be associated with stroke susceptibility [2, 7]. Importantly, emerging data suggest that many gene single nucleotide polymorphisms (SNPs) are correlated with different types of strokes [2, 7]. For example, rs11833579 of *WNK* and rs12425791 of *NINJ2* have been found closely associated with Asian stroke and ischemic stroke, respectively [11], and *ARHGEF10* gene polymorphism is closely associated with the risk of ischemic stroke in Northern Han Chinese population [12]. The next challenge would be to elucidate the pathological roles for these genes and SNPs in stroke.

*CONNEXIN37* gene (GJA4, also known as Cx37) encodes a gap junction protein. It has been reported that *CONNEXIN37* C1019T polymorphism results in the change of proline to serine [13], leading to deregulation of eNOS expression and functions [14]. Moreover, *CONNEXIN37* C1019T polymorphism has been revealed to affect endothelial function and elasticity leading to atherosclerosis [15]. Studies in different human races have demonstrated that *CONNEXIN37* gene polymorphisms are associated with coronary heart disease [16–19], which increases ACS mortality [20]. Several studies suggest that *CONNEXIN37* C1019T polymorphism may be also correlated to acute myocardial infarction [21–24] and subclinical arteries sclerosis indicators such as carotid artery thickness (IMT), arterial compliance and others [25–27]. A 10.7-year cohort study found that C1019T polymorphism of *CONNEXIN37* was associated with stroke [25], while another case-control study conducted in Taiwan showed C1019T polymorphism of *CONNEXIN37* was not related to Stroke [28]. Therefore, the association of *CONNEXIN37* polymorphism with stroke remains to be determined in Chinese and other populations.

In this study, we investigated the *CONNEXIN37* SNPs in 385 ischemic stroke patients and 362 hypertension patients without stroke.

## Methods

### Subjects

A cohort of 385 ischemic stroke patients and 362 control patients were enrolled in this study. The ischemic stroke group included 297 Han Chinese patients as diagnosed during the epidemiological investigation of Fuxin rural areas (Liaoning province, China) between 2004 and 2006 [29], and 88 diagnosed in our hospital. The diagnosis of these 385 ischemic patients was based on WHO standards of thrombotic patients with cerebral infarction and neurological abnormalities. The diagnosis of thrombotic cerebral infarction was confirmed by CT and/or MRI. The 362 control patients were from the same population of ischemic stroke group with matched gender and ethnic during the epidemiological survey. These control patients had hypertension with no history of stroke and other abnormalities, and did not undergo CT or MRI and other imaging studies. The stroke-free patients were controlled for smoking, drinking, blood sugar, cholesterol, obesity and other indicators during the selection [29]. The exclusion criteria were as follows: there was no blood relationship between the selected subjects and the selected subjects had no coronary heart disease, myocardial infarction, carotid and peripheral arterial stenosis, atrial arrhythmias, cancer, or trauma. The participants were not genetically related in three generations. After providing informed consent, 5 ml venous blood was obtained from each participant and kept at − 20 °C until analysis. This study was approved by the ethics committee of China Medical University.

### SNP selection and genotyping

Genomic DNA was extracted from EDTA anti-coagulated whole blood in Experimental Center and Virology Laboratory of Shengjing Hospital of China Medical University using TIANamp Blood DNA Kit (Tiangen Biochemical Technology, Beijing, China) according to manufacturer’s protocols. According to the previous literature and NCBI database, genotype detection was selected at the site of secondary allele frequency (MAF) > 0.1 in Beijing Chinese Han population. Tag SNP strategies were used to select the following two potential functional SNPs of *CONNEXIN37* gene from the dbSNP and HapMap databases: rs1764391 (ACCCACCCCCTCAGAATGGCCAAAAA[C/T]CCCCAAGTCGTCCCAGCAGCTCTGC); rs1764390 (GGCGAGTCAGTGTGGGGTGACGAGCA[A/G]TCAGATTTCGAGTGTAACACGGCCC). The minor allele frequency (MAF) of these SNPs was greater than 0.1 and pair-wise r^2^ was more than 0.8. Ligase detection reaction (LDR) method was used for genetyping [30–32]. Primers were designed with Primer 5 and probes were designed by Shanghai Generay Biotech (http://www.generay.com.cn/). The sequences of primers and probes are listed in Tables 1 and 2. Allele and genotype frequencies were determined by analyzing the raw data from ABI 3730XL with Peak Scanner Software v1.0 (ABI). To ensure quality control, genotyping was done without knowledge of case/control status of the subjects, and 10% random samples of cases and controls were genotyped twice by different persons; the reproducibility was 100%.

**Table 1:** PCR primer sequences used in this study

SNP	Primer name	Sequence 5′ to 3′
rs1764391	Forward	GTCTTCTTCTACCTCCCCGTG
rs1764391	Reverse	TTCTCAGGACCCCTCTGTTGG
rs1764390	Forward	TGACGGTGCTCTTCATCTTCC
rs1764390	Reverse	GGTGTGCTGACGAAGAGGAAC

**Table 2:** Probe sequences used in this study

SNP	Probe name	Sequence 5′ to 3′
rs1764391	TC	CCACCCCCTCAGAATGGCCAAAAAC
rs1764391	TT	TTTCCACCCCCTCAGAATGGCCAAAAAT
rs1764391	TR	p-CCCCAAGTCGTCCCAGCAGCTCTGC-FAM
rs1764390	TA	TTTTCGAGTCAGTGTGGGGTGACGAGCAA
rs1764390	TG	TTTTTTTCGAGTCAGTGTGGGGTGACGAGCAG
rs1764390	TR	p-TCAGATTTCGAGTGTAACACGGCCCTTT-FAM

### Statistical analysis

Statistical analyses were performed using SPSS for Windows software (version 13.0; SPSS, Chicago, IL, USA) and SHEsis platform. Data are presented as mean ± standard deviation (SD) with normal distribution by using the t test. Count data are presented as number of cases and percentage and analyzed using chi-square X^2^ test. Comparison and analysis of genotype and allele frequencies between cases and controls was performed using Pearson X^2^ test. The genotype frequencies were tested for Hardy-Weinberg equilibrium (HWE) using X^2^ test. SHEsis statistical analysis platform was used to compute haplotype frequencies and linkage disequilibrium coefficient (D’ and r^2^). Associations between gene polymorphisms and ischemic stroke risk were estimated by computing odds ratios (ORs) and 95% confidence intervals (95%CI) from unconditional logistic regression models. *P* < 0.05 was considered statistically significant. Multivariate logistic regression analysis were used after adjusting for age, systolic blood pressure, diastolic blood pressure, body mass index, smoking and drinking history, triglycerides, high density lipid cholesterol, low density lipid cholesterol and fasting plasma glucose to test the association of gene variation and ischemic stroke risk.

## Results

### Association between SNPs and ischemic stroke risk

The characteristics of 385 ischemic stroke cases and 362 controls with hypertension are summarized as previously reported [12]. The genotype distributions of *CONNEXIN37* rs1764391 and rs1764390 between cases and controls and their associations with ischemic stroke are summarized in Table 3. Recessive, additive and dominant models were applied to test the risk analysis of these variations in stoke patients and control subjects. The associations between these SNPs and IS were also analyzed after adjusting for hypertension diabetes mellitus, hyperlipidemia and other conventional confounding risk factors. The allele frequencies of *CONNEXIN37* gene polymorphisms in controls and cases and their associations with ischemic stroke are shown in Table 4.

**Table 3:** Genotype frequencies of *CONNEXIN37* gene polymorphisms in cases and controls and their associations with ischemic stroke

Genotype	IS group (*n*)	Control group (*n*)	OR (95%CI)^a^	*P* value	Adjusted OR (95%CI)^b^	*P* value
rs1764391						
CC	217	242				
CT	160	101	1.767 (1.297-2.407)	0.000	1.748 (1.149-2.658)	0.009
TT	8	19	0.470 (0.201-1.094)	0.080	0.173 (0.052-0.580)	0.004
Recessive			0.383 (0.166-0.886)	0.025	0.138 (0.042-0.459)	0.001
Additive			1.262 (0.976-1.632)	0.076	1.066 (0.754-1.508)	0.718
Dominant			1.561 (1.160-2.102)	0.003	1.428 (0.955-2.134)	0.082
rs1764390						
AA	76	100				
AG	223	196	1.497 (1.050-2.134)	0.026	1.522 (0.937-2.473)	0.090
GG	86	66	1.715 (1.106-2.657)	0.016	1.278 (0.697-2.344)	0.428
Recessive			1.290 (0.901-1.847)	0.164	0.950 (0.577-1.565)	0.841
Additive			1.317 (1.058-1.639)	0.014	1.149 (0.851-1.552)	0.364
Dominant			1.552 (1.104-2.182)	0.011	1.456 (0.913-2.321)	0.115

^a^Univariate analysis

^b^Adjusted OR (Covariates: age, systolic blood pressure, diastolic blood pressure, body mass index, smoking, drinking history, triglycerides, HDL cholesterol, LDL cholesterol, fasting blood glucose)

**Table 4:** Allele frequencies of *CONNEXIN37* gene polymorphisms in controls and cases and their associations with ischemic stroke

Allele	IS group (*n*)	Control group (*n*)	OR (95%CI)	*P* value
rs1764391				
C allele	594	585	1.00	–
T allele	176	139	1.247 (0.971-1.601)	0.083
rs1764390				
A allele	375	396	1.00	–
G allele	395	328	1.272 (1.038-1.559)	0.020

In the logistic regression models, compared with CC genotype of rs1764391, CT but not TT genotypes were associated with an increased risk of ischemic stroke (OR 1.767, 95%CI 1.297-2.407 for CT, *p* < 0.001; OR 0.470, 95%CI 0.201-1.094 for TT, *p* = 0.08, respectively). Under recessive and dominant models, rs1764391 was significantly associated with IS (OR 0.383, 95% CI 0.166-0.886, *p* = 0.025 and OR 1.561, 95% CI 1.160-2.102, *p* = 0.003, respectively). After adjustment for stroke risk factors, only under recessive model was rs1764391 associated with IS with OR 0.138 (95% CI0.042-0.459, *P* = 0.001). The T allele was not associated with ischemic stroke risk with OR of 1.247 (95% CI 0.971-1.601, *p* = 0.083). These data indicate that the T allele of rs1764391 is not a risk factor for stroke.

In the logistic regression models, compared with AA genotype of rs1764390, AG and GG genotypes were associated with an increased risk of ischemic stroke (OR 1.497, 95% CI 1.050-2.134 for AG, *p* = 0.026; OR 1.715, 95% CI 1.106-2.657, for GG, *p* = 0.016, respectively). Under additive and dominant models, rs1764390 was associated with IS with OR and 95% CI 1.317 (1.058-1.639), *p* = 0.014; 1.552 (1.104-2.182), *p* = 0.011, respectively. After adjustment for stroke risk factors, the associations did not existed with OR and 95% CI 1.149 (0.851-1.552), *P* = 0.364; 1.456 (0.913-2.321), *P* = 0.115, respectively. The G allele was associated with ischemic stroke risk with OR of 1.272 (95% CI 1.038-1.559, *p* = 0.02). These data indicate that the G allele of rs1764390 is a risk factor for stroke.

### Haplotype analysis

We next analyzed the distribution of haplotypes in the cases and controls. The haplotypes with frequencies greater than 0.03% are shown in Table 5. Three haplotypes were constructed in *CONNEXIN37* based on the SNPs of rs1764391 and rs1764390. After correcting the *P* value for multiple testing, we found C-A and T-G were significantly associated with IS with OR and 95% CI 0.813 (0.662-0.998), *P* = 0.048; 1.403 (1.080-1.822), *P* = 0.011, respectively.

**Table 5:** Frequency distribution of haplotypes of *CONNEXIN37* gene in cases and controls

Haplotype ^a^	IS group (rate)	Control group (rate)	OR (95%CI)	*P* value
C-A	367 (0.476)	375 (0.518)	0.813 (0.662-0.998)	0.048
C-G	227 (0.295)	210 (0.290)	0.996 (0.796-1.246)	0.972
T-G	168 (0.218)	118 (0.163)	1.403 (1.080-1.822)	0.011

^a^ Frequency of < 0.03 were not included in the analysis

### Hardy-Weinberg balance analysis

The observed genotype frequencies of two SNPs followed Hardy-Weinberg equilibrium among the controls (*P* > 0.05 for two SNPs). For rs1764391, the χ2 and *p* values were 3.67 and 0.06 for control group, and 12.26 and 0.0005 for the patient group; for rs1764391, the χ2 and *p* values were 3.098 and 0.078 for control group, and 9.76 and 0.001 for the patient group. These results indicate a balanced genetic and Mendel population.

### Linkage disequilibrium test

**Fig. 1:**
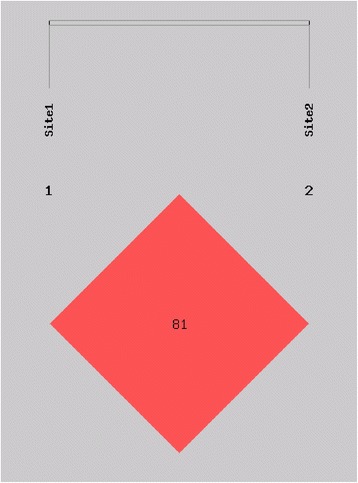
D′of the 2 SNPs. The SNPs rs1764391 and rs1764390 were in linkage disequilibrium

**Fig. 2:**
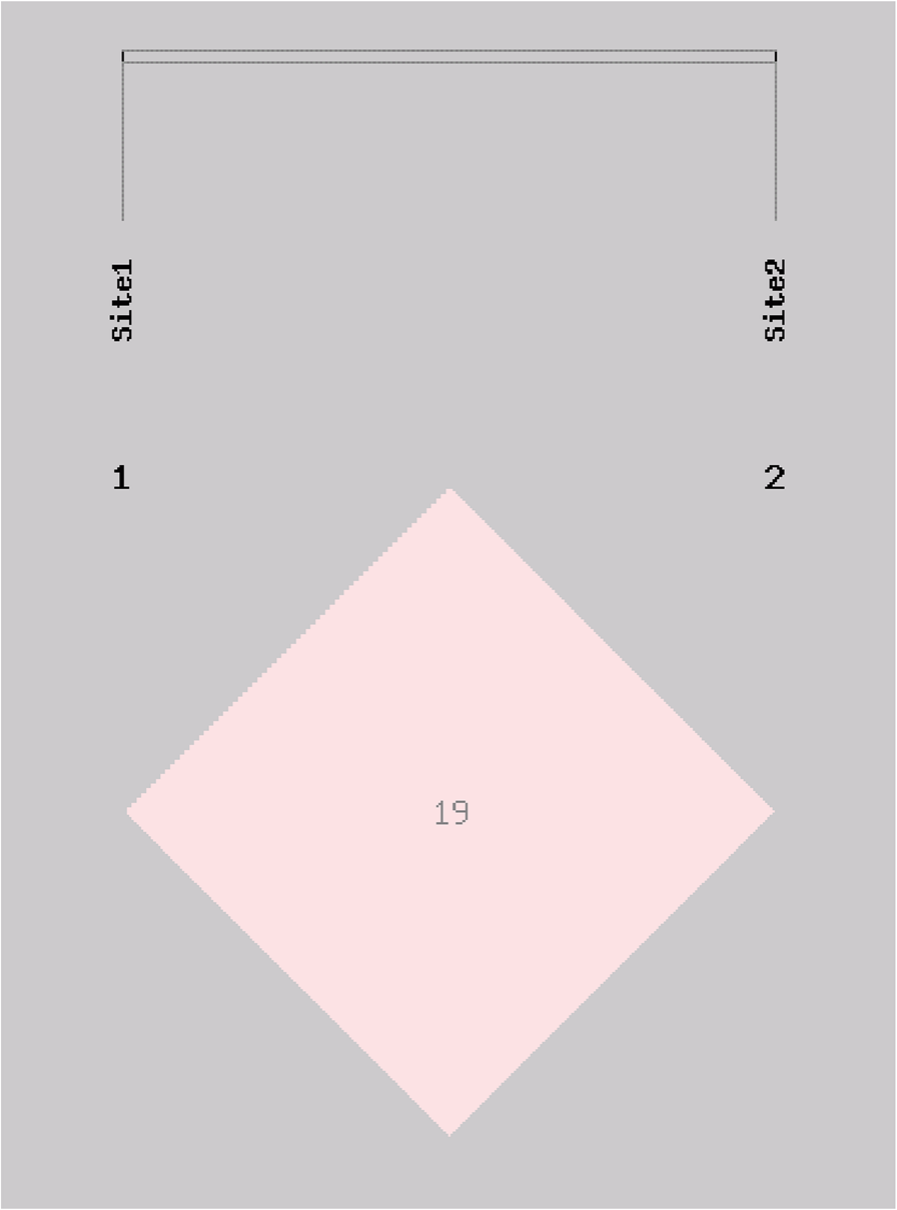
*r*2 of the 2 SNPs. The SNPs rs1764391 and rs1764390 were in linkage disequilibrium

SHEsis statistical analysis of the haplotype frequencies and linkage disequilibrium coefficient (D’ and r^2^) found that genotype frequencies of the two SNPs followed linkage disequilibrium but at a low level of Linkage Disequilibrium between these SNPs (Table 6 and Figs. 1 and 2).

**Table 6:** Linkage disequilibrium test

SNPs	D’	*r* ^2^
rs1764391- rs1764390	0.81	0.19

## Discussion

In this study, we found that AG and GG genotypes of SNP rs1764390 and CT genotype of rs1764391 in *CONNEXIN37* were associated with an increased risk of ischemic stroke, and that G allele of rs1764390 is a risk factor for ischemic stroke. We further demonstrated that SNP rs1764390 and SNP rs1764391 in *CONNEXIN37* were associated with ischemic stroke under additive/dominant model, and recessive/dominant model, respectively. To the best of our knowledge, this is the first study to demonstrate the association of SNPs rs1764390 and rs1764391 in *CONNEXIN37* with ischemic stroke risk in Chinese population.

Stroke is a late-onset disease with age and hypertension the independent risk factors. It has been shown that stroke rate is higher in higher blood pressure and older people [33, 34]. For these reasons, we selected places of residence and sex-matched, higher blood pressure, older patients without stroke as controls. As stroke is a disease of late onset, the selection of older patients as the control group may exclude the potential of genetic background of stroke. Except for obesity and other environmental factors that may be associated with stroke, the genetic factors may have greater impact on the proportion in young patients with relatively low blood pressure. Therefore, this study selected the baseline data that seem not match to highlight the genetic and other factors.

Stroke and coronary heart diseases are all arteriosclerosis-related diseases. It is thus possible that the two diseases may share common susceptible genes. Gap junction proteins are a series of transmembrane proteins that form the gap between cells and allow the exchange of ions and metabolites between cells. Connexin37 is a member of the gap junction protein family, which is mainly expressed in vascular endothelial cells, monocytes and macrophages [13]. These three kinds of cells are the most important cells involved in the arteriosclerosis process. Endothelial dysfunction is the first step of atherosclerosis, followed by migration of mononuclear cells to endothelial cells to form foam macrophages by the absorption of lipid components [35]. Arteriosclerotic mice have found that Connexin37 expression disappears in vascular endothelial cells and increases in foam macrophages [36]. This change in expression suggests that Connexin37 may be associated with arteriosclerosis. Accordingly, it was found that reducing the expression of Connexin37 in leukocytes could increase macrophage foam cells in atherosclerotic plaques, whereas knockout of *CONNEXIN37* exacerbates the aortic hardening plaques in apolipoprotein E-deficient mice (ApoE −/−). Wong et al. found that the expression of protein Cx37-pro319 (encoded by allele C) was less adherent than the expressed protein Cx37-ser319 (encoded by allele T) in mononuclear cell lines, suggesting that Connexin37 may inhibit mononuclear cells adhesion, the initiating factor of arteriosclerosis, to reduce atherosclerosis [16].

Previous studies on the *CONNEXIN37* gene were focused on rs1764391 locus (C1019T), where the changes in the T and C alleles result in the conversion of proline to tryptophan (P319S), which increases the incidence of myocardial infarction [23, 37]. The results of the study on the relationship between polymorphism and stroke in 2011 suggest that rs1764391 TT and CT (HR 2.83 and 1.69, respectively) have a higher risk ratio than the CC genotype. However, the results of previous studies on the relationship between the rs1764391 allele and atherosclerotic disease are not consistent. Some scholars believe that the two are related, but others think they are irrelevant. Most studies suggest that the C allele is more likely to lead to the occurrence of related diseases, while other studies suggest that the T allele is a causative gene [21, 23, 25, 37]. This difference may be related to the number of cases, but more likely to be associated with the genetic background of different populations. The NCBI database suggests that the frequency of alleles in Asians and Europeans is very different (T 0.151-0.636), indicating difference of the relationship between polymorphism and disease. Therefore, the interpretation of the results should consider different genetic background. Consistent with most previous studies, the results of the present study showed that the rs1764391 locus of the CT + TT and rs1764390 locus AG + GG genotypes was stroke-related, and allele G was the risk of stroke. Our study suggests that the *CONNEXIN37* gene mononucleotide polymorphism is associated with ischemic stroke in the northern Chinese Han population. Our findings have provided additional evidence of the importance of *CONNEXIN37* in human diseases, especially ischemic stroke.

There are some potential limitations of the present study. First, we only examined the rural elderly patients in Northern China, which may not present on behalf of the populations of other regions, ethnic or social background. Secondly, although Power and Sample Size Calculation (version 3.1.2, 2014) (http://Biostat.mc.vanderbilt.edu/wiki/Main/PowerSampleSize) revealed a power of 0.9 based on the lowest MAF among SNPs [38], the overall sample size was relatively small. Studies of multi-centered with a larger sample size from different regions, ethnic or social background are needed.

## Conclusion

Our findings have provided new insights into the roles of *CONNEXIN37* SNPs rs1764390 and rs1764391 in the development and progression of ischemic stroke risk in northern Chinese Han population.
